# Intra-beat biomarker for accurate continuous non-invasive blood pressure monitoring

**DOI:** 10.1038/s41598-022-19096-6

**Published:** 2022-10-06

**Authors:** Arash Abiri, En-Fan Chou, Chengyang Qian, Joseph Rinehart, Michelle Khine

**Affiliations:** 1 Department of Biomedical Engineering, University of California Irvine, Irvine, CA 92697 USA; 2 Department of Anesthesiology & Perioperative Care, University of California, Irvine Medical Center, Orange, CA USA

**Keywords:** Physiology, Cardiology, Health care, Nanoscience and technology

## Abstract

Accurate continuous non-invasive blood pressure (CNIBP) monitoring is the holy grail of digital medicine but remains elusive largely due to significant drifts in signal and motion artifacts that necessitate frequent device recalibration. To address these challenges, we developed a unique approach by creating a novel intra-beat biomarker (Diastolic Transit Time, DTT) to achieve highly accurate blood pressure (BP) estimations. We demonstrated our approach’s superior performance, compared to other common signal processing techniques, in eliminating stochastic baseline wander, while maintaining signal integrity and measurement accuracy, even during significant hemodynamic changes. We applied this new algorithm to BP data collected using non-invasive sensors from a diverse cohort of high acuity patients and demonstrated that we could achieve close agreement with the gold standard invasive arterial line BP measurements, for up to 20 min without recalibration. We established our approach's generalizability by successfully applying it to pulse waveforms obtained from various sensors, including photoplethysmography and capacitive-based pressure sensors. Our algorithm also maintained signal integrity, enabling reliable assessments of BP variability. Moreover, our algorithm demonstrated tolerance to both low- and high-frequency motion artifacts during abrupt hand movements and prolonged periods of walking. Thus, our approach shows promise in constituting a necessary advance and can be applied to a wide range of wearable sensors for CNIBP monitoring in the ambulatory and inpatient settings.

## Introduction

Blood pressure (BP) is an important physiologic metric that provides insights on a patient's cardiac function, volume status, organ perfusion, and overall hemodynamic stability^1^. Most commonly, BP is measured intermittently at the arm using a non-invasive sphygmomanometer (i.e., arm cuff). In high acuity medical settings, such as the operating room (OR) and intensive care unit (ICU), continuous BP monitoring is achieved via an invasive arterial catheter (A-line) placed in a peripheral artery^2^. While the A-line allows for the detection of sudden hemodynamic changes, since A-lines are highly invasive and associated with a number of medical complications, including hematoma, arterial thrombosis, and infection, their use is often limited to patients who are “high risk”^3–6^. In the U.S., it is estimated that only 36% of critically ill patients in the ICU receive an A-line^7^. Given the arm cuff’s relatively low precision compared to the A-line, and its tendency to overestimate low BPs and underestimate high BPs, its use as the sole instrument for measuring BP in most patients results in undetected critical hemodynamic changes that may have otherwise influenced patient care^8–11^. Additionally, recent studies have correlated continuous BP patterns with cardiovascular outcomes; for example, the variability in beat-to-beat BP measurements can be used to assess important physiological parameters, such as vascular tone, fluid responsiveness, and sympathetic autoregulation^12–15^.

For this reason, continuous noninvasive BP (CNIBP) monitoring has garnered increasing interest over the past several decades yet remains an elusive unmet need. One of the first discovered CNIBP techniques was the volume-clamp method, which has been implemented in several commercial devices, including ClearSight (Edwards Lifesciences, Irvine, CA, USA), Caretaker (Caretaker Medical NA, Charlottesville, VA, USA), and CNAP (CNSystems, Graz, Austria)^16–18^. Despite meeting the accuracy guidelines (mean average error ≤ ± 5 mmHg and standard deviation < 8 mmHg) cited by the Association of Advancement of Medical Instrumentation (AAMI) and U.S. Food and Drug Administration (FDA), their widespread use has been largely limited due to their bulkiness, high cost, and inconvenient form factor^19,20^.

Photoplethysmography (PPG)-based devices have attracted attention for their smaller form factor compared to volume-clamp devices^19^. As they are susceptible to interference from ambient light, skin tone, changes in applanation pressure, and low-frequency baseline wander, PPG-based devices rely on a number of pre-processing steps, and often include moving average filters, frequency filters, and other noise-reduction techniques, such as discrete wavelet transformation (DWT) or empirical mode decomposition (EMD)^21–26^. Furthermore, since PPG requires high applanation pressures to achieve adequate morphologic resolution, many devices have transitioned to using the PPG signal’s temporal dimension via pulse wave velocity (PWV) to more reliably estimate BP^20,27^. PWV can be calculated from either the pulse transit time (PTT) or pulse arrival time (PAT). The former is defined as the time taken by a pressure wave to travel between two arterial sites and can be calculated by using two synchronized PPG signals at two different peripheral sites. The latter is defined as the PTT interval plus the pre-ejection period, which represents the delay between electrical depolarization of the left ventricle and the onset of ventricular ejection^28,29^. By using an additional nearby electrocardiogram (ECG) device, PAT was developed to overcome the significant challenge of calibrating two anatomically distant PPG devices.

Many studies have described methods for estimating BP using PTT or PAT. Two of the most cited algorithms, developed under the basis of the Moens–Kortweg equation, were created by Chen et al. and Poon et al., and proposed quick calibration of PTT/PAT values by using a single reference BP value (e.g., arm cuff)^30–32^. While both studies had initially shown satisfactory correlations to the radial A-line, McCarthy et al. demonstrated that Chen’s algorithm actually required recalibration every 4 minutes and tracked BP changes poorly^33^. Furthermore, in the context of vasoactive drugs, Payne et al. suggested that the calibration interval be further reduced to every 60 beats^34^. Conversely, Poon’s algorithm demonstrated improved BP tracking capabilities; however, it required recalibration every 45 seconds to stay within AAMI standards^33^. Indeed, since their inception, numerous variations of these algorithms have been developed with the aims of improving accuracy and reducing calibration dependency^19–21^. Moreover, pulse wave decomposition analysis and more complex models (e.g., via machine learning) have been implemented to improve measurement performance^35,36^. However, the external validity of these algorithms and their ability to perform in different clinical contexts has been difficult to assess, since most studies do not report their calibration intervals and data’s hemodynamic ranges.

In 2016, Addison et al. proposed a single intra-beat PPG signal feature coined slope transit time (STT) as an alternative of PTT for tracking blood pressure^37^. While this approach was not validated against the A-line or an FDA-cleared device, a recent study demonstrated its ability to estimate systolic blood pressure (SBP) in the context of artificially generated baseline wander^38^. Most recently, Xu et al. proposed normalized STT (NSTT), which aimed to improve the stability of STT with normalization by PPG height^39^. Among 40 hemodynamically stable subjects, this approach demonstrated comparable results to an FDA-cleared tonometry device. However, since their technique relied on performing linear regression on their recorded waveform features, future investigation using clearly defined, distinct training and validation datasets are still needed in order to assess its accuracy.

The advent of Microelectromechanical System (MEMS) technology has introduced the prospect of using small, wearable capacitive pressure (CAP) sensors for CNIBP monitoring^40,41^. Over the years, CAP sensors have grown increasingly popular due to their convenient form factor, high spatial resolution, quick response times, and low power consumption requirements^40–42^. Unlike PPG- and oscillometry-based devices, CAP sensors detect pulsatile flow from the artery by measuring the changes in capacitance that result from compression and expansion of the soft dielectric layer. Thus, similar to arterial tonometry, CAP sensors are placed directly over the artery, and with the use of an initial arm cuff measurement, can be calibrated to measure beat-to-beat BP^43^. Although CAP sensors were previously limited by their low sensitivities, recent advancements in sensor design have largely overcome this challenge^20,44,45^. While studies have previously demonstrated the potential for CAP sensors to correlate well with measurements from the A-line, similar to PPG, quality of the data is highly dependent on applanation pressures^46^. Additionally, due to respiratory variations and the stochastic behavior of the viscoelastic polymer sensors, they are highly susceptible to baseline wander^47^. Thus, as with PPG signals, scientists have had to employ a variety of filters to eliminate this low frequency baseline wander. While seemingly successful, these filters have been primarily tested on short signal segments where there were little to no changes in BP. For example, studies have often employed high-pass frequency filters with cut-offs of 0.25–0.5 Hz to reduce baseline wander; however, this approach may impede the ability to perceive slow physiological drifts in BP that fall below this frequency range^21,48,49^. Moreover, studies have not examined how these filters may alter interpretations of blood pressure variability (BPV). Therefore, the validity and safety of using such filters on physiological signals that are intended to inform medical decisions remains largely unknown.

**Figure 1:**
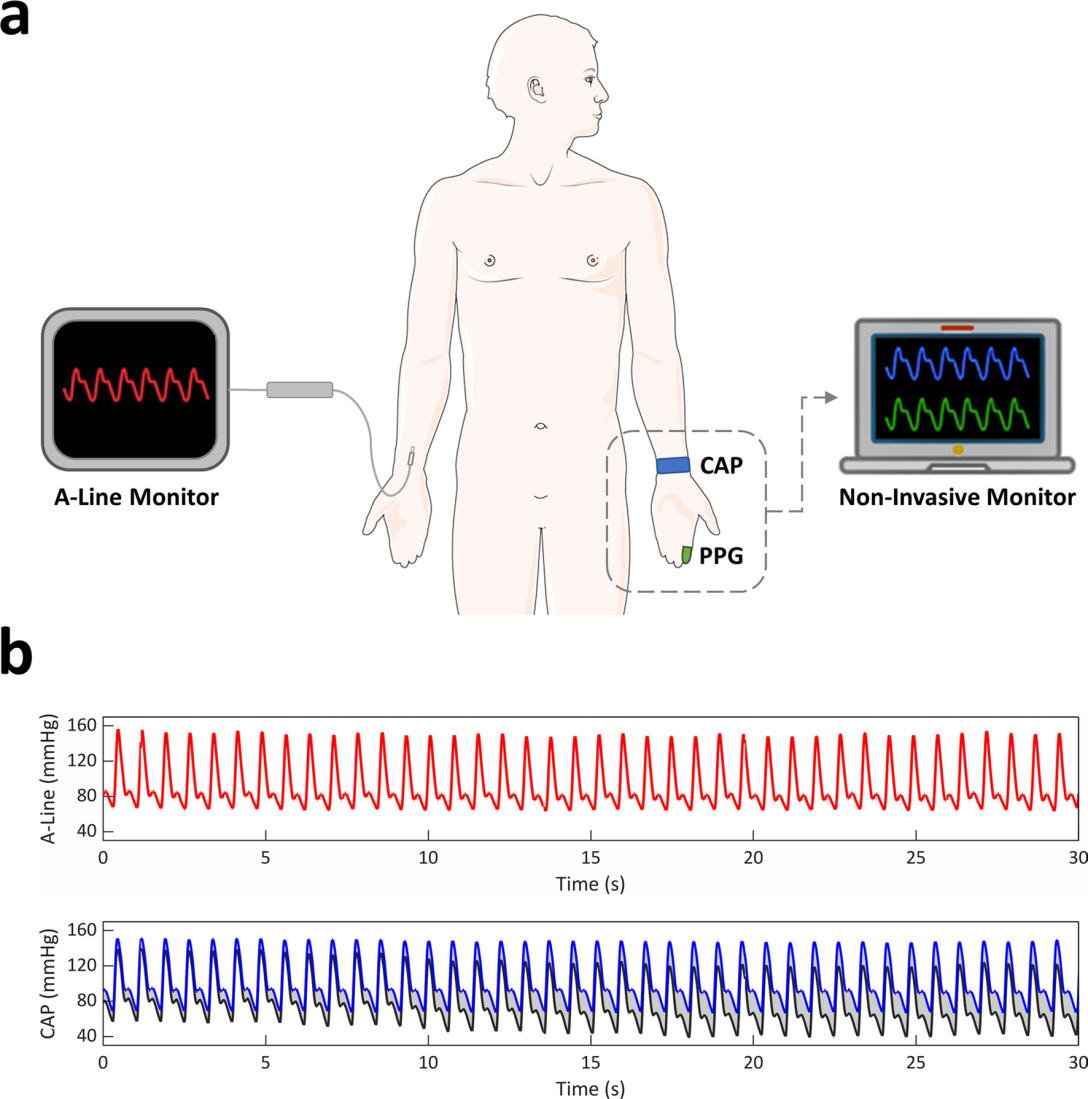
Overview of DTT approach. (**a**) Inpatient BP data was acquired using an invasive radial A-line and contralaterally-placed noninvasive CAP or PPG sensor. (**b**) BP signals from noninvasive sensors were processed using our DTT algorithm to obtain measurements comparable to those of the A-line. Gray shaded region indicates the offset between raw (gray line) and DTT-processed (blue line) signals.

In this study, we aimed to overcome the aforementioned shortcomings by developing a new intra-beat biomarker that we coined Diastolic Transit Time (DTT) to achieve highly accurate BP estimations. We defined DTT as the time from systolic peak to diastolic trough within a heartbeat. Unlike PTT or PAT that necessitate the use of multi-sensor systems, our algorithm utilizes the morphology of the hemodynamic waveform to enable single-sensor BP monitoring^21,32,50^. Compared to other commonly employed signal processing techniques, including bandpass filter (BF), DWT, and STT, we demonstrated our approach’s superior performance in eliminating stochastic baseline wander, while maintaining signal integrity and BP estimation accuracy in the context of significant (> 10% from baseline) hemodynamic changes. We applied this novel algorithm in a demographically and medically diverse cohort of 15 OR patients and showed that we could achieve high correlations between the adjusted non-invasive CAP sensor data and gold standard A-line BP measurements in the context of stress- and drug-induced hemodynamic perturbations for as long as 20 min without re-calibration (Fig. 1). Furthermore, we established our approach’s generalizability and ability to be applied to other waveforms by demonstrating its efficacy in correlating PPG waveforms obtained from ICU patients to A-line measurements. Using BPV as a spatiotemporal signal measure, we also verified that our algorithm did not significantly alter BP signal integrity. Moreover, as a proof-of-concept, we demonstrated that our algorithm could be applied to BPV analyses by identifying associations between beat-to-beat BPV and age, hypertension, and vascular disease. Finally, to establish our approach’s potential for future applications in ambulatory or outpatient monitoring, we examined its performance in the context of motion artifacts and demonstrated that, when applied to CAP sensor measurements, our DTT algorithm was able to compensate for baseline shifts from sudden arm/hand movements and walking.

In summary, our algorithm demonstrates several advantages to existing techniques, including: (1) enabling single-sensor BP monitoring, (2) removing stochastic baseline wander, (3) maintaining spatiotemporal signal integrity, (4) mitigating motion artifacts from sudden (e.g., hand movements) or cyclic (e.g., walking) movements, thereby (5) facilitating accurate BP measurements for patient monitoring. Thus, we introduce a unique and generalizable algorithm based on a novel intra-beat biomarker to correct raw BP data (i.e., continuous hemodynamic waveforms) obtained from various sensor sources to accurately estimate BP parameters, with significant implications in enhancing CNIBP technologies and overcoming challenges that have heretofore hindered their wide-scale adoption and usefulness.

## Results

### Participants

BP data was obtained from 15 surgical patients (Table 1), 10 of whom were female. For the OR cohort, the average age was 57.8 (range: 22–79) years and average BMI was 27.4 (range: 20.0–34.0) kg/m^2^. A total of 10,226 s of intra-operative BP recordings were extracted, with an average segment length of 204.5 (range: 60–1200) seconds. The ICU cohort consisted of 20 BP recordings, totaling 8405 s in duration, from different patients being treated at an ICU. The average segment length of the ICU recordings was 420.3 (range: 180–590) seconds. Due to the anonymized nature of the database, demographics were not available for the ICU patients.

**Table 1:** Surgical patient demographics.

Subject	Age	Sex	BMI	SBP Range^a^ (mmHg)	DBP Range^a^ (mmHg)	Procedure	Comorbidities
1	24	F	24.0	88–108	41–56	Open abdominal surgery	None
2	74	M	34.0	126–157	69–81	Microvascular decompression	AF, bigeminy
3	78	F	33.5	97–122	42–53	Laparoscopic abdominal surgery	Asthma
4	23	F	20.0	84–97	41–50	Right calf sarcoma resection	None
5	55	F	32.0	86–109	51–67	Open abdominal surgery	HTN
6	62	M	30.7	98–133	49–63	Radical cystoprostatectomy	HTN, smoker
7	79	F	25.0	126–158	73–92	Open abdominal surgery	HTN
8	61	F	27.0	108–124	51–66	Pancreaticoduodenectomy	T2DM
9	64	F	29.0	91–153	47–79	Pancreaticoduodenectomy	None
10	22	F	30.2	90–106	50–62	Hip surgery	None
11	72	M	26.6	79–148	34–61	Open abdominal surgery	None
12	52	F	23.0	93–134	47–71	Ankle surgery	None
13	61	F	21.6	102–143	45–58	Pancreaticoduodenectomy	VD
14	61	M	23.0	102–138	52–76	Open abdominal surgery	None
15	79	M	31.0	126–155	53–69	Open abdominal surgery	HTN

*BMI* body mass index, *SBP* systolic blood pressure, *DBP* diastolic blood pressure, *AF* atrial fibrillation, *HTN* hypertension, *T2DM* type 2 diabetes mellitus, *VD* vascular disease.

^a^Blood pressure was measured from an arterial line and ranges are representative of the recordings that were tested in this study.

### Comparing algorithm performance

**Figure 2:**
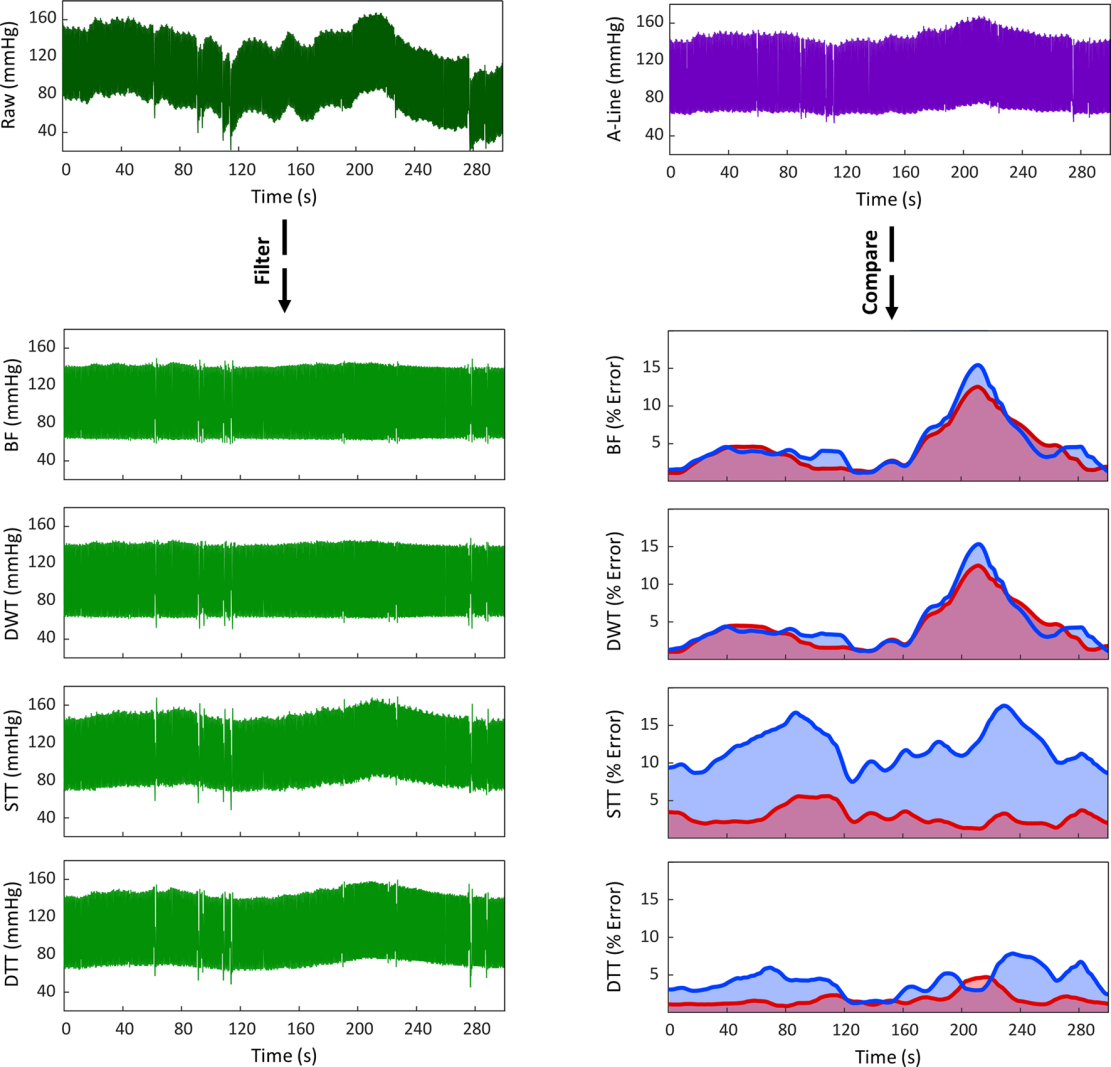
Evaluating algorithm accuracy and BP tracking ability. (left) A 300-s BP recording from the CAP sensor was processed using four different methods: bandpass filter (BF), discrete wavelet transformation (DWT), slope transit time (STT), and diastolic transit time (DTT). (right) BP estimates from the processed signals were compared to A-line measurements to assess errors in SBP (red line) and DBP (blue line).

To compare our algorithm’s accuracy, calibration dependency, and BP tracking ability with those of other commonly employed methods, we utilized a 300-s segment of CAP sensor and A-line BP recordings from a hemodynamically unstable surgical patient (Fig. 2). Based on our review of the literature, we selected to compare our algorithm to 3 common methods: bandpass filtering (BF) using a 4th order Chebyshev II filter with cutoff frequencies of 0.5 and 10 Hz^23,45^, reduction of 7th level approximation coefficients using DWT with Daubechies 4 wavelets^51,52^, and BP estimation using the STT approach^37,38^. When applied to the raw BP signal, our DTT algorithm demonstrated average SBP and DBP errors of 1.69% ± 0.85 and 4.15% ± 1.70, respectively. In contrast, average SBP errors were found to be significantly higher using the BF (4.48% ± 3.07), DWT (4.70% ± 3.51), and STT (2.80% ± 1.10) algorithms (all *p* < 0.001). Moreover, average DBP errors were higher using the DWT (4.38% ± 3.06, *p* = 0.048) and STT (11.98% ± 2.50, *p* < 0.001) methods. While measurement errors from the DTT algorithm were consistently below 8%, significant deviations in accuracy were observed with the other algorithms during hemodynamic changes (e.g., increasing BP), with errors surpassing 15%.

### Inpatient BP monitoring

**Figure 3:**
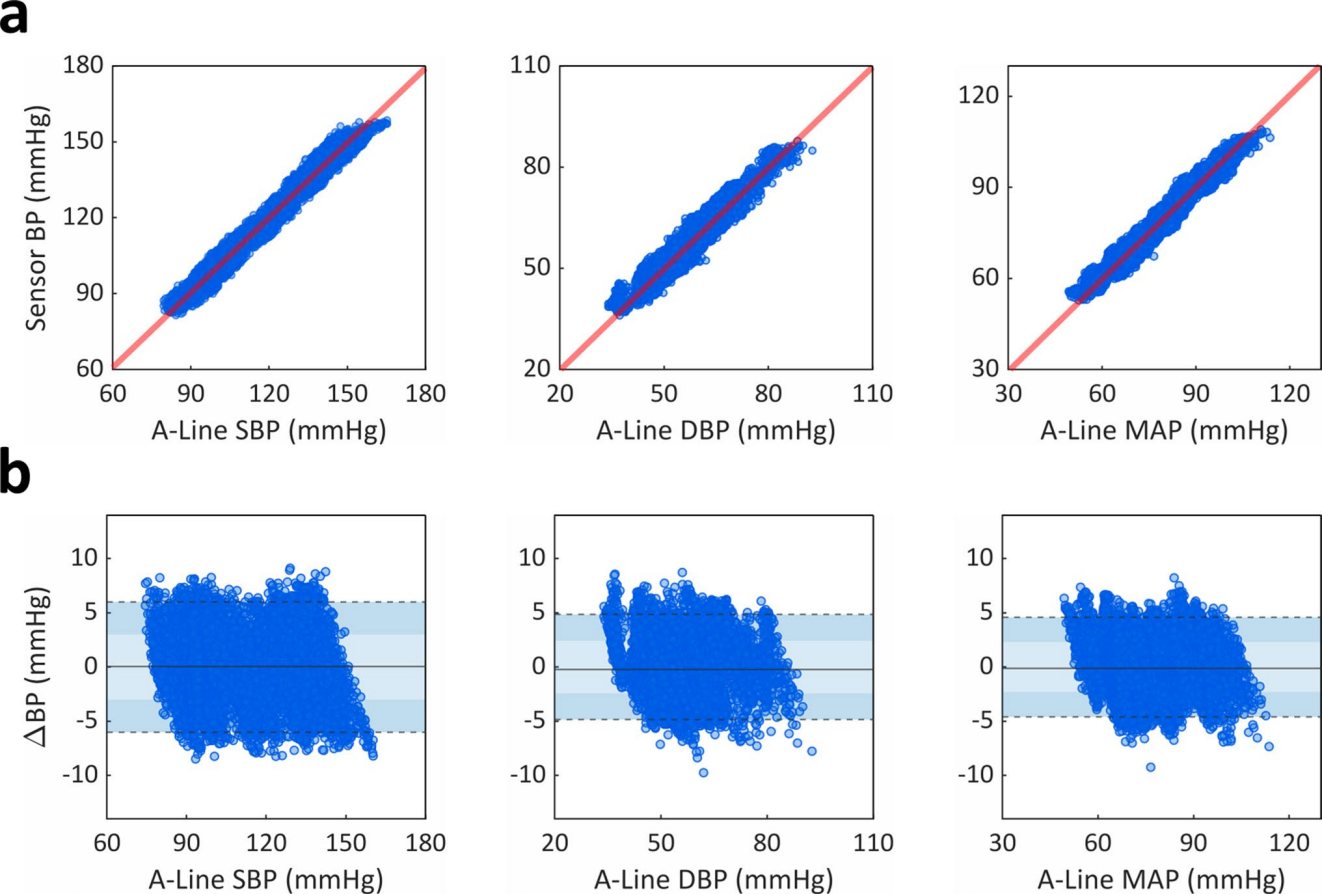
Assessing accuracy and precision of inpatient BP monitoring using a CAP sensor. (**a**) Pearson correlation and (**b**) Bland–Altman analyses compared beat-to-beat (left) SBP, (middle) DBP, and (right) MAP measurements between the noninvasive CAP sensor and invasive A-line. (**a**) CAP sensor measurements (n = 11,002) showed strong linear correlations to the A-line, with Pearson coefficients of 0.987, 0.960, and 0.980 for SBP, DBP, and MAP, respectively. (**b**) CAP sensor measurements (n = 11,002) demonstrated mean biases of 0.05 (3.07), − 0.21 (2.47), and − 0.12 (2.35) mmHg for SBP, DBP, and MAP, respectively. Light- and dark-shaded areas represent 68% (1 SD) and 95% (1.96 SD) limits of agreement, respectively.

**Figure 4:**
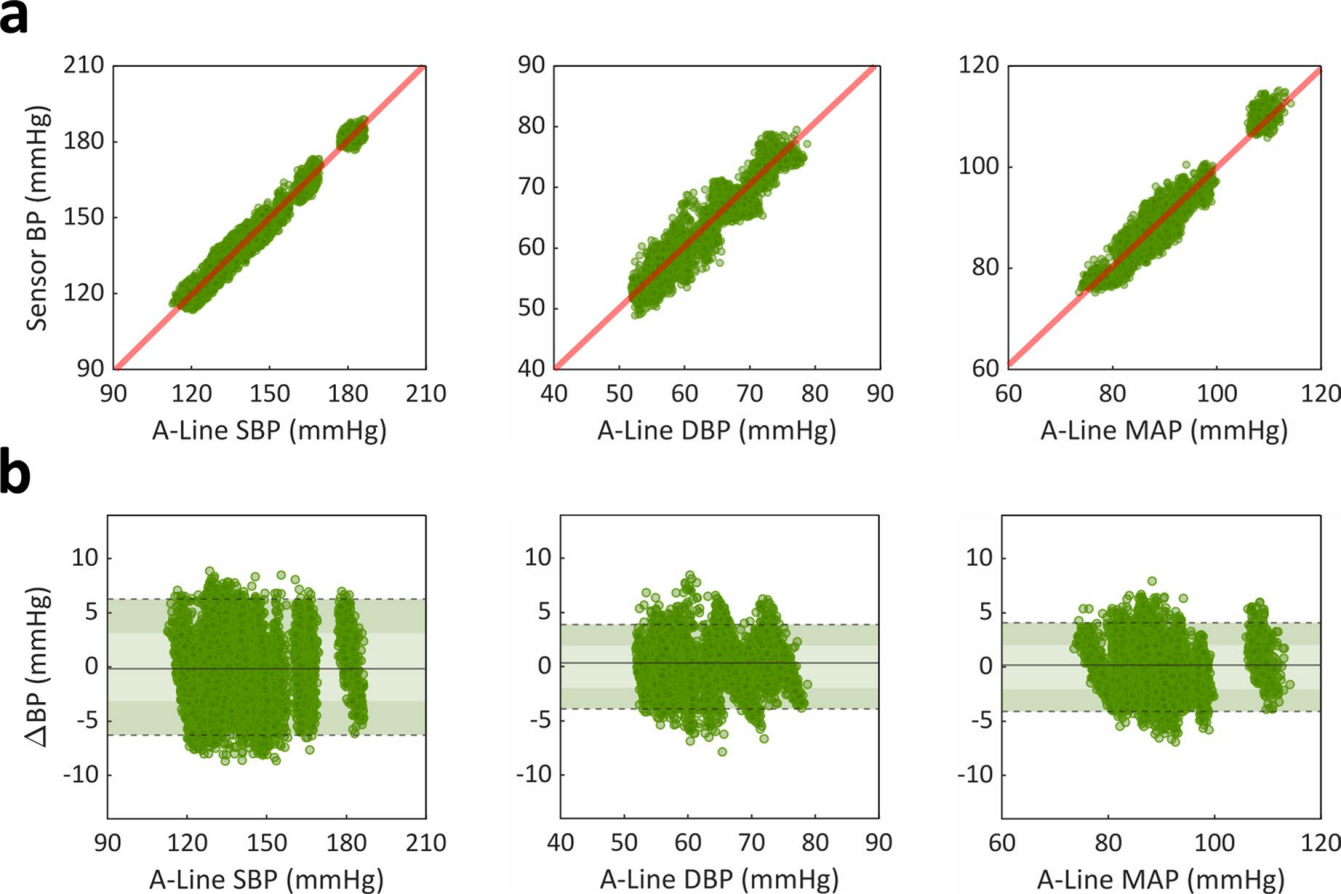
Assessing accuracy and precision of inpatient BP monitoring using a PPG sensor. (**a**) Pearson correlation and (**b**) Bland–Altman analyses compared beat-to-beat (left) SBP, (middle) DBP, and (right) MAP measurements between the noninvasive PPG sensor and invasive A-line. (**a**) PPG sensor measurements (n = 9628) showed strong linear correlations to the A-line, with Pearson coefficients of 0.982, 0.958, and 0.952 for SBP, DBP, and MAP, respectively. (**b**) PPG sensor measurements (n = 9628) demonstrated mean biases of − 0.14 (3.20), 0.36 (1.99), and 0.19 (2.09) mmHg for SBP, DBP, and MAP, respectively. Light- and dark-shaded areas represent 68% (1 SD) and 95% (1.96 SD) limits of agreement, respectively.

Using BP recordings from high acuity patients (OR and ICU), we evaluated our algorithm’s ability to correct raw BP signals and accurately measure critical cardiovascular parameters (Figs. 3 and 4). Overall, our algorithm was applied across a wide hemodynamic range: SBP 79–158 mmHg, DBP 34–92 mmHg, MAP 49–115 mmHg, HR 44–123 beats per minute (bpm).

In the OR cohort, we demonstrated strong linear correlations between CAP sensor estimations and gold standard A-line measurements, with Pearson coefficients of 0.987, 0.960, and 0.980 for SBP, DBP, and MAP, respectively (Fig. 3a). The resulting mean bias (SD) for SBP, DBP, and MAP were 0.05 (3.07), − 0.21 (2.47), and − 0.12 (2.35) mmHg, respectively (Fig. 3b). Additionally, HR measurements from the CAP sensor strongly agreed with those from the A-line (mean bias: 0.02 ± 1.53 bpm). This was a stark improvement from measurements obtained without using our DTT algorithm, which exhibited mean biases (SD) of − 6.63 (16.30), − 6.56 (16.35), and − 6.59 (16.20) for SBP, DBP, and MAP, respectively.

To demonstrate the generalizability of the DTT algorithm to other modalities, we further evaluated the accuracy of our approach by applying it to PPG measurements obtained from ICU patients. We similarly showed strong linear correlations to the A-line, with Pearson coefficients of 0.982, 0.958, and 0.952 for SBP, DBP, and MAP, respectively (Fig. 4a). Moreover, we demonstrated high estimation accuracies, with mean bias (SD) of − 0.14 (3.20), 0.36 (1.99), and 0.19 (2.09) mmHg for SBP, DBP, and MAP, respectively (Fig. 4b). Additionally, HR measurements from the PPG sensor strongly agreed with those from the A-line (mean bias: − 0.02 ± 1.58 bpm). This was once again a significant improvement in accuracy compared to measurements obtained without using our DTT algorithm, which exhibited mean biases (SD) of − 1.71 (7.20), − 1.12 (5.20), and − 1.32 (5.08) for SBP, DBP, and MAP, respectively.

### BPV as a predictor of cardiovascular health

Assessment of beat-to-beat BPV can provide unique perspectives on important cardiovascular parameters and physiologic states. Thus, to verify that our approach maintained BPV integrity, we applied our DTT algorithm to the gold standard A-line measurements for all 15 OR patients and evaluated if BPV would be significantly altered. On average, the SDs of processed and unprocessed A-line measurements were 1.35 and 1.40 mmHg for SBP, 0.97 and 0.90 mmHg for DBP, and 1.02 and 1.01 mmHg for MAP, respectively. Overall, there was no statistically significant difference in BPV between these two groups for SBP (*p* = 0.351), DBP (*p* = 0.272), and MAP (*p* = 0.426).

**Figure 5:**
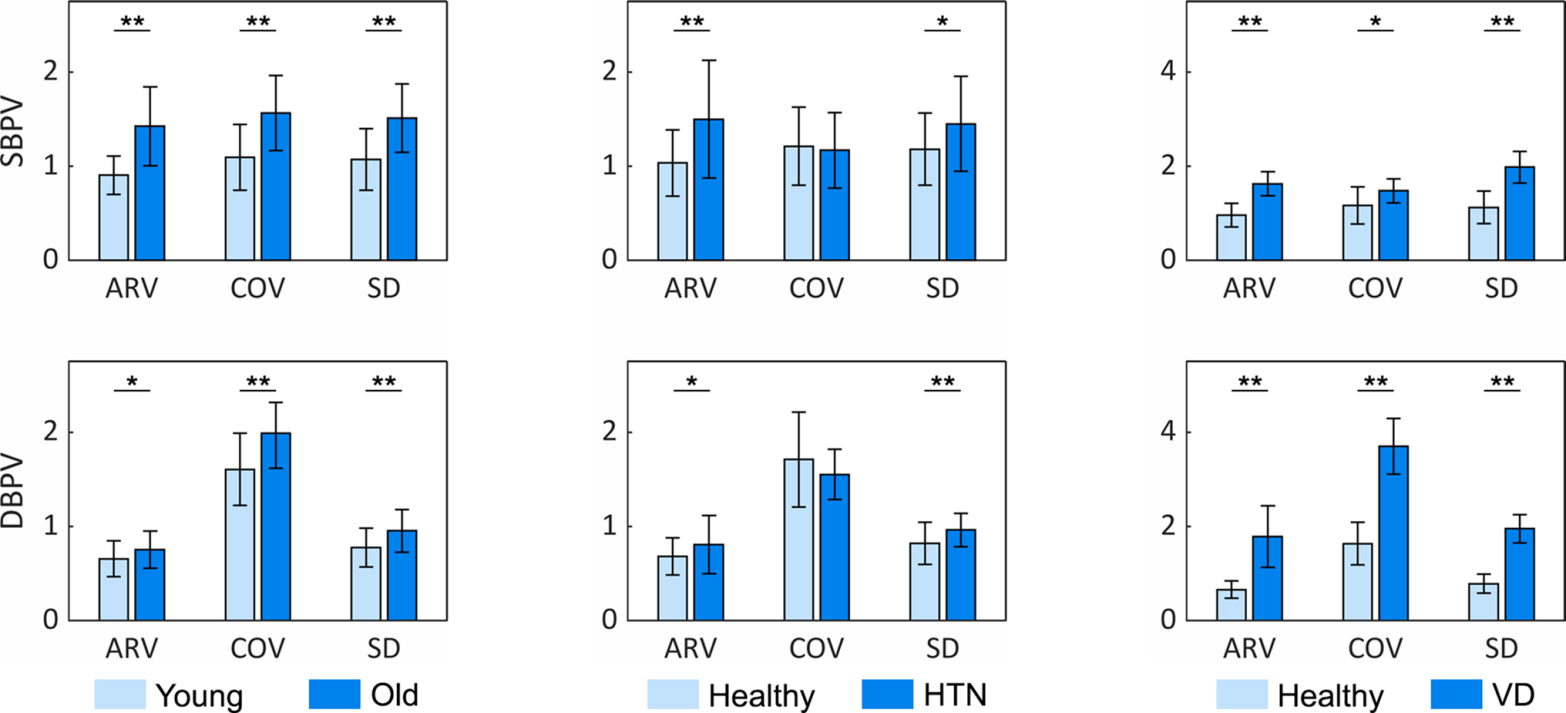
Associations between BPV and patient demographics. OR patients (n = 15) were stratified by age, history of hypertension (HTN), and history of vascular disease (VD). BPV was assessed using three metrics: standard deviation (SD), coefficient of variation (COV), and average real variability (ARV). *signifies *p* < 0.05; ** signifies *p* < 0.01.

Hence, to uncover associations between BPV and cardiovascular health, OR patients were stratified into cohorts according to their age, history of hypertension, and history of vascular disease (VD). The systolic (SBPV) and diastolic BPV (DBPV) of subjects were subsequently evaluated and compared across groups (Fig. 5). In the age-stratified cohort, older subjects (> 60 years old) were found to have significantly higher SBPV and DBPV than younger subjects (< 30 years old) across all variability indices (all *p* < 0.05, Supplementary Table S1). On the other hand, hypertensive patients demonstrated significantly higher SD and average real variability (ARV) than healthy patients for both SBP and DBP (all *p* < 0.05). Finally, vascular disease was found to be associated with a significantly higher SBPV and DBPV (all *p* < 0.05).

### Ambulatory BP monitoring

**Figure 6:**
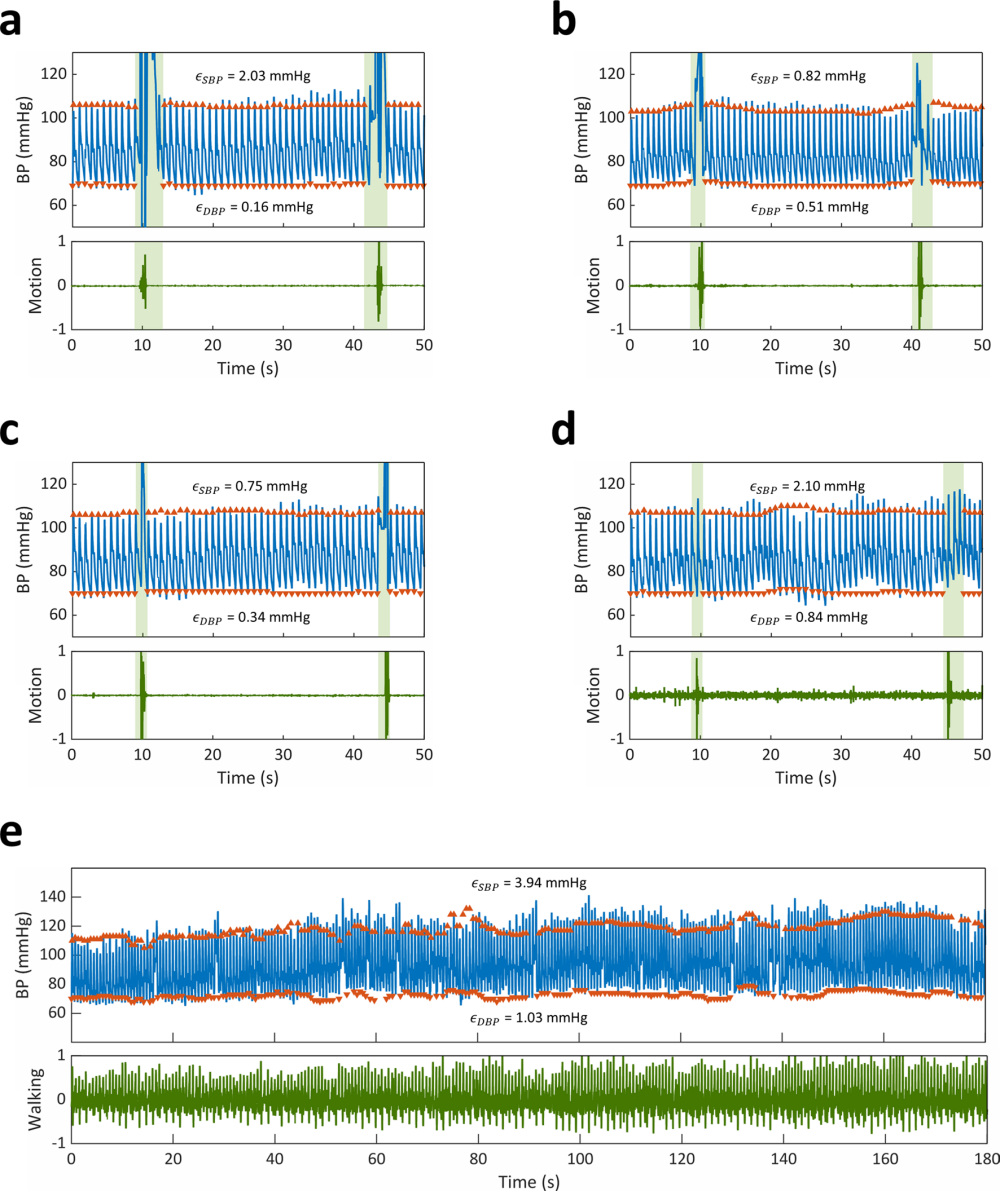
Measurement performance in ambulatory BP monitoring. CAP sensor (blue line) and Caretaker (orange arrows) BP measurements with corresponding normalized accelerometer signal (green line) during (**a**) 180° wrist rotation, (**b**) 90° wrist flexion, (**c**) hand closure, (**d**) wrist hit/impulse, and (**e**) walking. (**a**–**d**) Green shaded regions indicate periods of movement. Upward and downward facing arrows represent Caretaker SBP and DBP, respectively.

Intermittent motion artifacts present a significant challenge for ambulatory and outpatient CNIBP. To investigate our algorithm’s potential for future implementation into these settings, we assessed its performance in correcting CAP sensor measurements in the context of common arm/hand movements − 180° wrist rotation, 90° wrist flexion, hand closure, and wrist hit/impulse—and walking (Fig. 6). Movements were tracked using an accelerometer embedded in the CAP sensor’s wireless board, which was attached to the subject’s hand using an elastic strap. Motion was represented by the normalized magnitude (− 1 to 1) of the accelerometer’s measurements.

Overall, the processed CAP sensor signals exhibited excellent agreement with Caretaker measurements. On average, the mean biases (SD) were 2.03 (2.55), 0.82 (2.83), 0.75 (2.6), and 2.10 (3.60) mmHg for SBP and 0.16 (2.09), 0.51 (1.86), 0.34 (2.16), and 0.84 (3.73) mmHg for DBP during wrist rotation, wrist flexion, hand closure, and wrist hit/impulse, respectively. Additionally, over the duration of three minutes of walking, our algorithm’s BP estimates agreed well with Caretaker measurements and demonstrated mean biases of 3.94 (5.49) and 1.03 (3.23) mmHg for SBP and DBP, respectively.

## Discussion

In this study, we demonstrated the efficacy of our novel DTT approach in eliminating stochastic baseline wander to achieve accurate beat-to-beat BP measurements, well-within the limits demanded by AAMI/ISO standards, using noninvasive CAP and PPG sensor recordings from surgical and ICU patients. Despite their many advancements over the years, CNIBP monitors (e.g., PPG, tonometry) all continue to face significant baseline drift and noise that prevent accurate, long-term continuous BP measurements^53^. For example, Kaisti et al. recently developed a non-invasive wearable MEMS pressure sensor that demonstrated remarkable temporal and morphological BP waveform accuracy; however, they were unable to measure BP amplitude due to reported baseline variations^54^. Some devices have attempted to circumvent this issue by requiring recalibration as frequent as every minute^46,55,56^. Naturally, this has made such devices often impractical and has stymied their adoption into clinical practice. Therefore, our algorithm’s ability to effectively remove this problematic drift and overcome this long-existing challenge can have significant implications in bringing existing and future CNIBP monitoring technologies closer to clinical use. Additionally, unlike many methods that require a posteriori signal processing, such as filters or regressions, or machine learning-based approaches that are computationally expensive and require training on large datasets, our DTT approach is algorithmically simple and may be more adaptable to real-time monitoring applications.

Since a number of cardiovascular changes occur at varying BPs and heart rates, it is important for measurement techniques to be validated across a wide hemodynamic range before being considered sufficiently reliable for use in clinical practice^53^. Thus, by using a heterogenous and hemodynamically labile cohort of patients for our analyses, we were to evaluate our algorithm’s performance across a wide range of physiologic states and establish its validity for clinical use. However, nearly 52% of prior studies have reportedly used only normotensive patients for testing, severely limiting their external validity. Moreover, only 4% of studies have included hypotensive patients and fewer than 8% have included hypertensive patients in their analyses^57^. Therefore, this study serves as one of a select series of reports that have investigated CNIBP monitoring accuracy across a representative cohort of patients.

The ability for an algorithm to accurately track BP changes is also an important dynamic measure that is rarely assessed. Studies have frequently utilized pre-processing methods, such as BF and DWT, to reduce noise and baseline drift, with seemingly acceptable results^21,23–26^. However, since these methods are commonly applied to healthy, young subjects and their results reported as aggregate averages, it is often not possible to characterize their behaviors in the context of active BP changes. By utilizing a hemodynamically labile segment of BP measurements, we were able to characterize and compare our approach’s ability to track BP changes with other common techniques. As hypothesized, the accuracies of BF- and DWT-based methods faltered during changes in BP. This is likely due to the filters’ inability to distinguish between artificial (i.e., noise) and physiological BP drift, since the basis of both methods rely on signal frequency without considering individual waveform characteristics. On the other hand, STT-based BP estimation exhibited slightly higher errors in SBP, but significantly greater deviations in DBP than our approach. As the BP waveform can be affected by many factors, it is possible that the STT method’s reliance on a single systolic feature limited its ability to the reflect changes in both SBP and DBP^39^.

Validation experiments indicated that our DTT algorithm did not significantly alter BP signal variability, suggesting that our approach may be applied to future studies on short-term BPV. Our proof-of-concept experiments supported this conclusion, as we were able to identify several interesting associations between short-term BPV and cardiovascular health. Our findings demonstrated that older age was associated with increased SBPV and DBPV, in agreement with other studies that have suggested it to be due to increased arterial stiffness and impaired baroceptor function^58,59^. Additionally, we observed that patients with hypertension exhibited higher ARVs and SDs for SBP and DBP than healthy individuals. This is consistent with Xia et al. findings, which suggested that hypertensive patients may possess compromised vascular elasticity, and hence elevated BPV^60^. While we did not observe a significant difference in coefficient of variation (COV) between the two groups, this may have been attributed to a type II error from a limited sample size or due to COV’s lower sensitivity for short-term changes than ARV^61,62^. Finally, we demonstrated a significantly higher SBPV and DBPV in vascular disease patients than healthy individuals. Interestingly, there was an observably larger inter-group difference in DBPV than SBPV. This was likely a result of the characteristic increase in arterial wall stiffness associated with vascular disease^12,63^.

As the demand increases for wearable devices that enable continuous day-to-day biomonitoring, significant efforts have been made towards developing systems that support long-term ambulatory recording. However, despite many advancements in CNIBP monitoring technologies over the years, ambulatory BP monitoring continues to be an elusive undertaking. The presence of motion-related artifacts and abrupt changes in signal baseline introduce an especially complex confounding factor in BP estimation algorithms that make accurate and precise ambulatory BP monitoring a significant challenge^48,53^. While several promising techniques have been recently developed, compensating for motion artifacts, especially those from “macro-motions” during walking or jogging, that possess an overlapping frequency spectrum with the BP signal remains a challenge^48,64,65^. In our experiments, we showed that our DTT algorithm was able to recover quickly from sudden baseline shifts caused by abrupt hand/arm movements. Additionally, we demonstrated our algorithm’s high tolerance to low- (e.g., arm swing) and high-frequency (e.g., step impulse) motion artifacts through its ability to accurately measure beat-to-beat BP during a prolonged period of walking. Importantly, these strong correlations were achieved without using any frequency filters or advanced signal processing techniques, as evidenced by the remnant signs of walking-induced enveloping in the DTT-processed BP signals. While additional testing during more intense activities is warranted, our DTT approach, nonetheless, shows potential as a means towards bringing CNIBP monitoring to the ambulatory setting.

Although our approach overcomes several critical obstacles in CNIBP monitoring, there are still limitations that warrant future study. While our DTT algorithm mitigates much of the challenges associated with baseline wander and motion artifacts, as with existing CNIBP technologies^48,49,66,67^, it is still susceptible to the confounding effects incurred by changing applanation pressure. Quantitative characterization of pulse waveforms shows promise as a method of automatically assessing signal quality and detecting perturbations in applanation^68^. Several groups have also demonstrated the feasibility of using a secondary pressure sensor or force transducer as a feedback mechanism within their system to account for changes in contact pressure^69–71^. For the purposes of demonstrating our DTT approach, we manually excluded segments of data with visibly low-quality waveforms and utilized a moving median filter as a coarse method to remove remaining intermittent disruptions in signal quality. Future iterations of our algorithm will benefit from a machine learning classification model (e.g., support-vector machine, logistic regression) to automatically assess signal quality and more precisely exclude corrupt waveforms^72,73^. Additionally, while we tested our algorithm against a wide and dynamic range of cardiovascular parameters, further testing of this technique against a larger sample of patients would better elucidate its generalizable efficacy. Moreover, future investigations are warranted to directly compare the accuracy and tracking performances of our DTT algorithm with multi-sensor approaches using PTT or PAT. Nonetheless, our DTT approach shows promise in constituting a necessary advance for wearable sensors for CNIBP monitoring in the ambulatory and inpatient settings.

## Methods

### Data acquisition

BP data was acquired from subjects in the OR, ICU, and ambulatory settings. The OR cohort consisted of 15 intraoperative patients receiving treatment at the University of California, Irvine (UCI) Medical Center between June 2020 and March 2021. All patients were under general anesthesia and received intravenous medications (e.g., ephedrine, phenylephrine) whenever clinically indicated. BP recordings from surgical patients were simultaneously obtained invasively via radial A-line and noninvasively using a CAP sensor placed on the contralateral radial artery (Fig. 1a). BP data for the ICU cohort was obtained from a public database (UCI Machine Learning Repository) and consisted of 20 randomly-selected recordings of invasive radial A-line and non-invasive radial PPG measurements collected from patients being treated at ICU facilities^74,75^. Ambulatory BP measurements used for motion artifact analysis were obtained from one healthy subject using an FDA-cleared CNIBP monitoring device (Caretaker; Caretaker Medical NA, Charlottesville, VA, USA) and a noninvasive CAP sensor placed at the contralateral radial artery. Informed consent was obtained from all OR and ambulatory subjects, and experimental protocols were approved by and conducted in accordance with the UCI Institutional Review Board (IRB no. 2019-5251 and 2016-2924). Usage of the ICU recordings was IRB exempt due to the anonymized and deidentified nature of the public database.

### Signal quality assessment and pre-processing

All collected BP signals were pre-processed in MATLAB (R2021a, The MathWorks, Natick, Massachusetts, USA) prior to analysis. Using the devices’ integrated clocks, noninvasive BP measurements (via CAP or PPG sensors) were synchronized with the recordings from reference devices (A-line or Caretaker) to a precision of one second. Next, BP recordings were manually determined by the authors to exclude sections of data with low-quality signal based on the following criteria: (1) signals were at least 30 s in length and (2) consisted of at least 30 s of continuous BP waveforms possessing clearly visible systolic peaks, dicrotic notches, and diastolic troughs. Since inconsistent applanation pressure is a major source of measurement error in CNIBP monitors^48,49^, and manual manipulation (e.g., repositioning) of sensors could not be controlled for in the OR and ICU setting, an unsupervised algorithm was developed to objectively identify and exclude segments of data that contained significant deviations in applanation. Since perturbations in contact pressure alter signal amplitude, a change in BP waveform contractility, defined as the maximum of the first derivative of the systolic upstroke, was used as a surrogate marker of changing applanation. Hence, for a given pair of noninvasive and invasive BP signals, regressions of the change in normalized contractility were calculated and compared using a hypothesis test. The sensors were considered to have significant deviations in applanation if the pair of regressions were statistically different (*p* < 0.05). A 30-s sliding window was utilized in this step to minimize the amount of excluded data.

### Signal correction and BP estimation

To utilize as an input for our algorithm, BP data must consist of continuous (i.e., beat-to-beat) hemodynamic waveforms, as shown in Fig. S1. As an initial step, we used the *BP_annotate* package in MATLAB to identify the peaks and troughs in our raw BP signal and extract each BP waveform^76^. Once extracted, these waveforms were used by our algorithm to calculate beat-to-beat DBP based on an initial calibration measurement and each waveform’s distinct morphology (Eq. 1). These “raw” waveforms were subsequently transformed into units of pressure (Eq. 2) and baseline-corrected using the calculated DBP values. The algorithm’s steps are further detailed below.

Using an initial BP measurement from a standard BP cuff, beat-to-beat diastolic blood pressure (DBP) was calculated as a function of DTT, defined as the time from systolic peak to diastolic trough, and waveform contractility (Fig. S1):

1 $$eDBP\left( t \right) = SBP_{0} - \left[ {m_{0} *DTT\left( t \right)*\left( {\frac{C\left( t \right)}{{C_{0} }}} \right)^{ - 1} } \right]$$

where *eDBP* was the estimated DBP, *t* was time, *SBP*_*0*_ was the initial SBP cuff measurement, *m*_*0*_ was a calibration factor, and *C* was waveform contractility. The factor, m_0_, was calculated as an average of the first five recorded beats:

2 $$m_{0} = \frac{1}{5}*\mathop \sum \limits_{i = 1}^{5} \frac{{PP_{0} }}{{PP_{s} \left( i \right)}}*\frac{{SBP_{s} \left( i \right) - DBP_{s} \left( {i + 1} \right)}}{DTT\left( i \right)}$$

where *PP*_*0*_ was the initial pulse pressure obtained by cuff measurement, *PP*_*s*_ was the sensor pulse pressure, *SBP*_*s*_ was the sensor SBP, and *DBP*_*s*_ was the sensor DBP.

Thus, the calibration factor, m_0_ [mm Hg/s], served two purposes: (1) to convert the raw signal into pressure measurements [mm Hg] and (2) to track changes in DBP relative to the initial DTT. Beat-to-beat change in contractility was included as a dynamic transformation factor to adjust for stress- or drug-induced physiological changes in left ventricular contractility that can modify contraction and relaxation times and, consequently, alter BP waveform morphology^77,78^.

Since our algorithm calculated DBP using intra-beat parameters that were independent of the confounding effects introduced by low-frequency noise, by comparing the estimated DBP to raw DBP, we were able to empirically model the baseline wander in our recordings, which served as an offset for the raw BP signals (Fig. 1b). The calculated baseline wander was smoothed using a 30-point moving median filter before being subtracted from the raw BP signal. The corrected signal was then used to extract beat-to-beat DBP, SBP, and mean arterial pressure (MAP, Eq. 3). Outlier measurements were excluded using a 30-point moving median filter.

3 $$MAP = DBP + \frac{1}{3}*\left( {SBP - DBP} \right)$$

### Evaluating beat-to-beat BP variability

Since there is currently no well-established standard for measuring BPV, short-term (beat-to-beat) SBPV and DBPV were quantified using three different metrics: SD, COV, and ARV. SD is the most commonly used index and represents the global fluctuation of BP measurements around the mean^79^. COV is a normalized measure of SD and was defined by dividing by the mean BP^80^. ARV, which aims to account for the temporal order of measurements and reduce the errors produced by signal noise, was defined by the mean of the absolute differences between adjacent BP measurements^80–82^. Each BPV measurement represented an average over a 30-beat window. Since intraoperative infusion of vasoactive medications could artificially increase BPV, this confounding factor was mitigated by excluding BPV measurements from segments that exhibited BP ranges exceeding 10 mmHg.

### Statistical analysis

All statistical analyses were performed in MATLAB. A *p*-value less than 0.05 was considered statistically significant. A t-test or Wilcoxon signed rank test was used for continuous variables to evaluate differences between the means of two samples. Shapiro–Wilk tests were used to assess for normality. Brown–Forsythe tests were used to determine statistical differences in BPV between two sets of measurements. Pearson linear correlation coefficients were calculated to assess how well beat-to-beat noninvasive sensor measurements correlated with those of the A-line. Mean bias, SD, and 95% confidence intervals (CIs) were also calculated, which in combination with the Bland–Altman method of paired measurements, were used to assess agreement between noninvasive (CAP or PPG sensor) and invasive (A-line) BP monitoring methods. The benchmark for acceptance was based on AAMI/ISO 81060-2 standards (mean bias: 5 ± 8 mmHg), which are used for FDA clearance of non-invasive sphygmomanometers^83,84^.

## Supplementary Information

Supplementary Information 1.

Supplementary Information 2.

## Data Availability

The data that support the findings of this study are available from the corresponding author (M.K.) upon reasonable request.
