# Supplementary material for: Intra-beat biomarker for accurate continuous non-invasive blood pressure monitoring
Source: Sci Rep. 2022 Oct 6;12:16772. doi: 10.1038/s41598-022-19096-6 (PMC9537243; doi:10.1038/s41598-022-19096-6)
Supplement: Supplementary file 1 — Supplementary Information 1. [file 41598_2022_19096_MOESM1_ESM.docx]

**Supplementary Information**


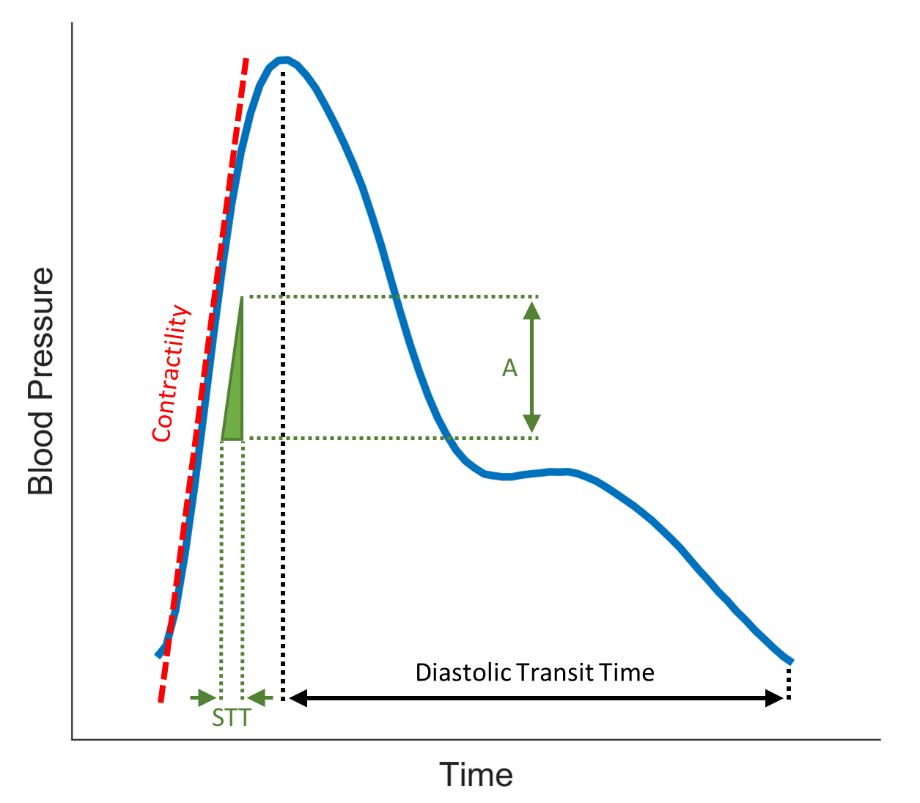


**Figure S1.** Plot of a representative blood pressure waveform (blue line) along with its corresponding waveform contractility (red dashed line), diastolic transit time, and slope transit time (STT; in respect to a constant amplitude A).
