# Supplementary material for: Intra-beat biomarker for accurate continuous non-invasive blood pressure monitoring
Source: Sci Rep. 2022 Oct 6;12:16772. doi: 10.1038/s41598-022-19096-6 (PMC9537243; doi:10.1038/s41598-022-19096-6)
Supplement: Supplementary file 2 — Supplementary Information 2. [file 41598_2022_19096_MOESM2_ESM.docx]

**Supplementary Materials**

| **Supplementary Table 1:** Measures of ARV, COV, and SD for age-, hypertension-, and vascular disease-stratified surgical patients. | | | | | | | | | | |
| --- | --- | --- | --- | --- | --- | --- | --- | --- | --- | --- |
|  | | **Age (n=8)** | | | **Hypertension (n=12)** | | | **Vascular Disease (n=8)** | | |
|  |  | **Young** | **Old** | ***P*** | **Healthy** | **HTN** | ***P*** | **Healthy** | **VD** | ***P*** |
| **SBP** | **ARV** | 0.906 (0.203) | 1.426  (0.419) | <0.001 | 1.036  (0.352) | 1.500  (0.625) | 0.002 | 0.962  (0.252) | 1.628  (0.255) | <0.001 |
|  | **COV** | 1.094  (0.349) | 1.566  (0.398) | <0.001 | 1.212 (0.414) | 1.170  (0.402) | 0.716 | 1.168  (0.396) | 1.482  (0.255) | 0.013 |
|  | **SD** | 1.072  (0.326) | 1.512  (0.363) | <0.001 | 1.182  (0.384) | 1.450  (0.503) | 0.047 | 1.128  (0.346) | 1.981  (0.338) | <0.001 |
| **DBP** | **ARV** | 0.657  (0.191) | 0.755  (0.198) | 0.015 | 0.682  (0.196) | 0.817  (0.309) | 0.048 | 0.660  (0.185) | 1.788  (0.656) | <0.001 |
|  | **COV** | 1.607  (0.482) | 1.991  (0.470) | 0.003 | 1.711  (0.503) | 1.554  (0.265) | 0.384 | 1.637  (0.452) | 3.706  (0.593) | <0.001 |
|  | **SD** | 0.778  (0.207) | 0.954  (0.224) | 0.003 | 0.821  (0.223) | 0.963  (0.177) | 0.006 | 0.788  (0.199) | 1.953  (0.301) | <0.001 |
| *SBP: Systolic Blood Pressure; DBP: Diastolic Blood Pressure; HTN: Hypertension; VD: Vascular Disease; ARV: Average Real Variability; COV: Coefficient of Variation; SD: Standard Deviation*  Values reported as mean (SD). | | | | | | | | | | |
